# Swiss community-based surveillance of respiratory virus co-occurrence in 2022–2024

**DOI:** 10.1128/spectrum.03088-25

**Published:** 2026-03-30

**Authors:** Francisco Javier Pérez-Rodríguez, Ana Rita Goncalves Cabecinhas, Patricia Suter-Boquete, Tania Spedaliero, Nicolas Hulo, Laurent Kaiser, Isabella Eckerle, Manel Essaidi-Laziosi

**Affiliations:** 1 Geneva Centre for Emerging Viral Diseases, Geneva University Hospitals and University of Geneva, Geneva, Switzerland; 2 Laboratory of Virology, Department of Diagnostics, Geneva University Hospitals, Geneva, Switzerland; 3 Swiss National Reference Centre for Emerging Viral Infections, Geneva University Hospitals 27230 , Geneva, Switzerland; 4 Swiss National Reference Centre for Influenza, Geneva University Hospitals 27230 , Geneva, Switzerland; 5 Service for Biomathematical and Biostatistical Analyses, University of Geneva, Institute of Genetics and Genomics, Geneva, Switzerland; 6 Division of Infectious Diseases, Geneva University Hospitals 27230 , Geneva, Switzerland; 7 Department of Medicine, Faculty of Medicine, University of Geneva, Geneva, Switzerland; 8 Department of Microbiology and Molecular Medicine, Faculty of Medicine, University of Geneva, Geneva, Switzerland; Barnard College, Columbia University, New York, New York, USA

**Keywords:** respiratory viruses, co-occurrence, co-detection, interference, virus-virus interaction and surveillance

## Abstract

Viral respiratory infections (VRIs) are a persistent global health concern, driven by the year-round circulation of diverse viruses. During the coronavirus disease 2019 (COVID-19) pandemic, the prevalence of most respiratory viruses declined sharply following nonpharmaceutical interventions. Their subsequent resurgence and co-circulation with Severe Acute Respiratory Syndrome Coronavirus 2 (SARS-CoV-2) created a novel and unpredictable VRI landscape. We investigated the respiratory virus dynamics and co-occurrence patterns in symptomatic outpatients in Switzerland, using nasopharyngeal specimens collected between August 2022 and July 2024 by the National Reference Center for Influenza. In total, 5,369 specimens were tested by PCR or RT-PCR for 16 respiratory viruses. Co-detections were identified in 9% of positive samples and occurred year-round. The majority involved two viruses (92%), although triple and quadruple detections were also observed. Statistical analysis showed that multiple detections were significantly more common in children. Their incidence was higher not only with the most prevalent ones, rhinoviruses (RVs) and SARS-CoV-2, but also with bocaviruses (hBoV) and adenoviruses (ADVs). A statistically significant decrease in viral load was observed when a second virus was co-detected with SARS-CoV-2 or respiratory syncytial virus (RSV), but not with influenza viruses. This effect was most pronounced in early- and middle-aged adults. Through the systematic investigation of viral co-occurrence, this study emphasizes the importance of community-based surveillance. Beyond monitoring respiratory disease burden, such surveillance provides a foundation for generating hypotheses to better understand viral interactions and the virus-host interplay during infection.

**IMPORTANCE:**

Viral respiratory infections (VRIs) are a major public health challenge due to their constant circulation and significant impact on vulnerable populations. The coronavirus disease 2019 (COVID-19) pandemic profoundly altered the dynamics of respiratory viruses, leading first to a sharp decline in their prevalence, followed by an unpredictable resurgence in co-circulation with Severe Acute Respiratory Syndrome Coronavirus 2 (SARS-CoV-2). In this context, understanding viral interactions and their co-detection in the community has become essential. This project provides novel insights into the frequency and characteristics of viral co-infections in a real-world outpatient setting. It highlights age-related differences and demonstrates statistically significant effects on viral load, particularly for SARS-CoV-2 and respiratory syncytial virus (RSV). These findings underscore the importance of community-based surveillance, not only to monitor the overall burden of respiratory diseases but also to generate new hypotheses on virus-host interactions. Ultimately, this knowledge can guide the development of more effective prevention and management strategies.

## INTRODUCTION

Viral respiratory infections (VRIs) constitute a global health concern (1) and are caused by a large variety of viruses belonging to different families. These viruses can infect the upper and/or lower respiratory tracts, leading to clinical manifestations that range from mild to severe and even fatal outcomes. The pathogenesis of VRIs depends on viral characteristics, host factors, and virus-host interactions. According to the World Health Organization, VRIs are among the leading causes of morbidity and mortality worldwide, affecting especially at-risk populations such as children, older adults, and immunocompromised individuals. Monitoring respiratory viruses’ circulation constitutes, hence, an important public health topic and is the subject of many national and global surveillance programs (2).

Studies of VRI seasonality have highlighted environmental parameters and human behavior as the main determinants of epidemics’ occurrence in a cyclic fashion (3–5). Annual oscillation of respiratory infection outbreaks depends also on the nature of viruses (reviewed in reference 6). While a few viruses, like rhinoviruses (RVs), adenoviruses (ADV), and bocaviruses (hBoV), circulate all year round, a number of enveloped viruses including influenza A/B (IAV/IBV) and respiratory syncytial virus (RSV) are usually detected only in winter (7–9). Conversely, non-rhinovirus enteroviruses’ (EV) prevalence seems rather increased in summer (10). In 2020, the coronavirus disease 2019 (COVID-19)-related pandemic caused by the newly emerging Severe Acute Respiratory Syndrome Coronavirus-2 (SARS-CoV-2) was marked by a drastic drop in the overall prevalence of endemic respiratory viruses due to the nonpharmaceutical intervention measures implemented to contain viral transmission and mitigate the unprecedented sanitary crisis of the modern era. However, when the gradual relaxation of control measures started, we witnessed a progressive VRI resurgence, albeit with some unexpected temporal shift (reviewed in reference 11). One notable observation refers to the off-seasonal RSV activities reported in the northern and southern hemispheres in 2021 (12, 13). Like RSV, influenza activity had also spiked in unusual interseasonal periods in the Southern Hemisphere (14). The absence of influenza infection during the first year of COVID-19 has been followed by a longer incidence in the 2021/2022 and 2022/2023 fall periods (15, 16), with double peaks per season and probable extinction of the IBV/Yamagata lineage (17). The mechanisms behind and impact of the recent rebound of respiratory viruses, including SARS-CoV-2 activity, are not well understood. With the introduction of the novel SARS-CoV-2 alongside the existing respiratory viruses, the post-pandemic situation was unique and unpredictable and could be a beginning of a reshaping of global VRI dynamics (18, 19). Here, based on nation-wide surveillance data of the Swiss National Reference Center of Influenza (NRCI), we describe (i) the circulation pattern of common respiratory viruses over a 2-year period post-pandemic from July 2022 to August 2024 and (ii) investigate differences in viral loads between single and co-detections.

## MATERIALS AND METHODS

### Data

The Swiss outpatient sentinel surveillance system (Sentinella) that monitors influenza and other respiratory viruses (20) involves around 170 medical doctors in Switzerland, including general practitioners/internists and pediatricians (about 85% and 15%, respectively). The NRCI recommends the collection of nasopharyngeal swabs by participating physicians. These specimens, collected from primary care settings and/or domiciliary assistance, are weekly tested by the NRCI based at Geneva University Hospitals in Switzerland. The inclusion criteria were patients presenting with influenza-like illness (ILI) or acute respiratory infection (ARI) during consultations. Outcomes from this surveillance program are published by the NRCI on its website weekly and in annual reports and by the Federal Office of Public Health (FOPH) in its infectious diseases dashboard.

Conducted under the strict conditions defined in the data transfer and use agreement with the FOPH (DTUA V1.2.05.05.202), our study reviewed molecular diagnostic data of anonymized NRCI clinical samples collected and tested during the period from the week 31 of 2022 (w31/22) to the week 30 of 2024 (w30/24). Except for the age, the sex, the sampling week, and the (RT-)PCR result, no other data of the patients have been included in this study.

For this study, the pediatric population was defined as individuals under 16 years. This threshold was chosen based on physiological and epidemiological considerations as older adolescents resemble adults in immune response and disease presentation, ensuring a clear separation between pediatric and adult groups. Several studies have also used 16 years as the upper limit for pediatric cohorts (21–23).

### Sample testing

Nucleic acids were extracted from nasopharyngeal swabs using an automated extraction system, NucliSENS e-MAG (bioMérieux, Geneva, Switzerland) or QiaSyphony (Qiagen, Zug, Switzerland), and spiked with standardized canine distemper virus (CDV).

Multiplex testing using semi-quantitative real-time PCRs or reverse transcription (RT-)PCRs was performed using QuantStudio 5 and 7 Pro Applied Biosystems thermocyclers with the following temperature cycling conditions: 48°C for 30 min, 95°C for 3 min, and 40 cycles of 95°C for 10 s and 60°C for 1 min. The amplification assays use specific commercially produced custom mixes (Kaneka Eurogentec SA, Seraing, Belgium) consisting of sets of primers and probes designed according to *in silico* alignments or modified from publicly available combinations. Eight different mixes allowed (Eurogentec, see Table S1) to detect a panel of 15 respiratory viruses including 2 DNA viruses, hBoV and ADV, and 13 RNA viruses, namely, IAV, IBV, RSV, human metapneumovirus (hMPV), RVs, parainfluenza viruses 1, 2, 3, and 4 (PIV-1,-2, −3 and −4, respectively), and the four common cold coronaviruses (CoV-HKU1, HCoV-229E, HCoV-OC43, and HCoV-NL63). RV RT-PCR assay cross-detects the non-rhinovirus EVs with lower sensitivity, from the same family of picornaviruses. SARS-CoV-2 was separately tested either using Cobas SARS-CoV-2 targeting E and ORF1 genes (Roche, Switzerland) or Xpert Xpress SARS-CoV-2 targeting E and N genes (Cepheid, Switzerland). No housekeeping host gene was tested for normalization as a sample-quality control. However, the extraction efficiency was evaluated through the use of a spiked internal control, CDV (see Table S1).

Specimen’s viral loads were inferred from cycle threshold (CT) values of the respective assays. CT values are inversely correlated with viral load. For the sake of sensitivity differences between the different kits during the assessment of co-detection impact on SARS-CoV-2 viral load (VL), only samples tested with E genes Cobas (used for more than 97% of the SARS-CoV-2-positive samples) were analyzed. We defined co-infection/co-detection as the prevalence of samples with two or more respiratory viruses present and detected by (RT-)PCR.

### Statistical analyses

The age group-specific and virus-specific likelihoods of virus co-detection were assessed using 2 × 2 contingency tables with Fisher’s exact test and represented by the estimated odds ratio (OR) and 95% confidence interval (CI). For multiple testing, *P*-values were adjusted using the false discovery rate method.

For the examination of viral loads based on RT-PCR data, the normal distribution of the CT values was first evaluated using the Shapiro test. Accordingly, parametric (normal distribution) or otherwise nonparametric tests were performed. Statistically significant differences were examined using *t*-test or Wilcoxon test, and the correlation was analyzed using Pearson or Spearman tests.

For all these tests, the significance level was set at *P*-value < 0.05. All data sorting, statistical analyses, and graphs were performed in/obtained by R (version 4.3.2).

## RESULTS

### Circulation of respiratory viruses in Switzerland 2022–2024

**Fig 1:**
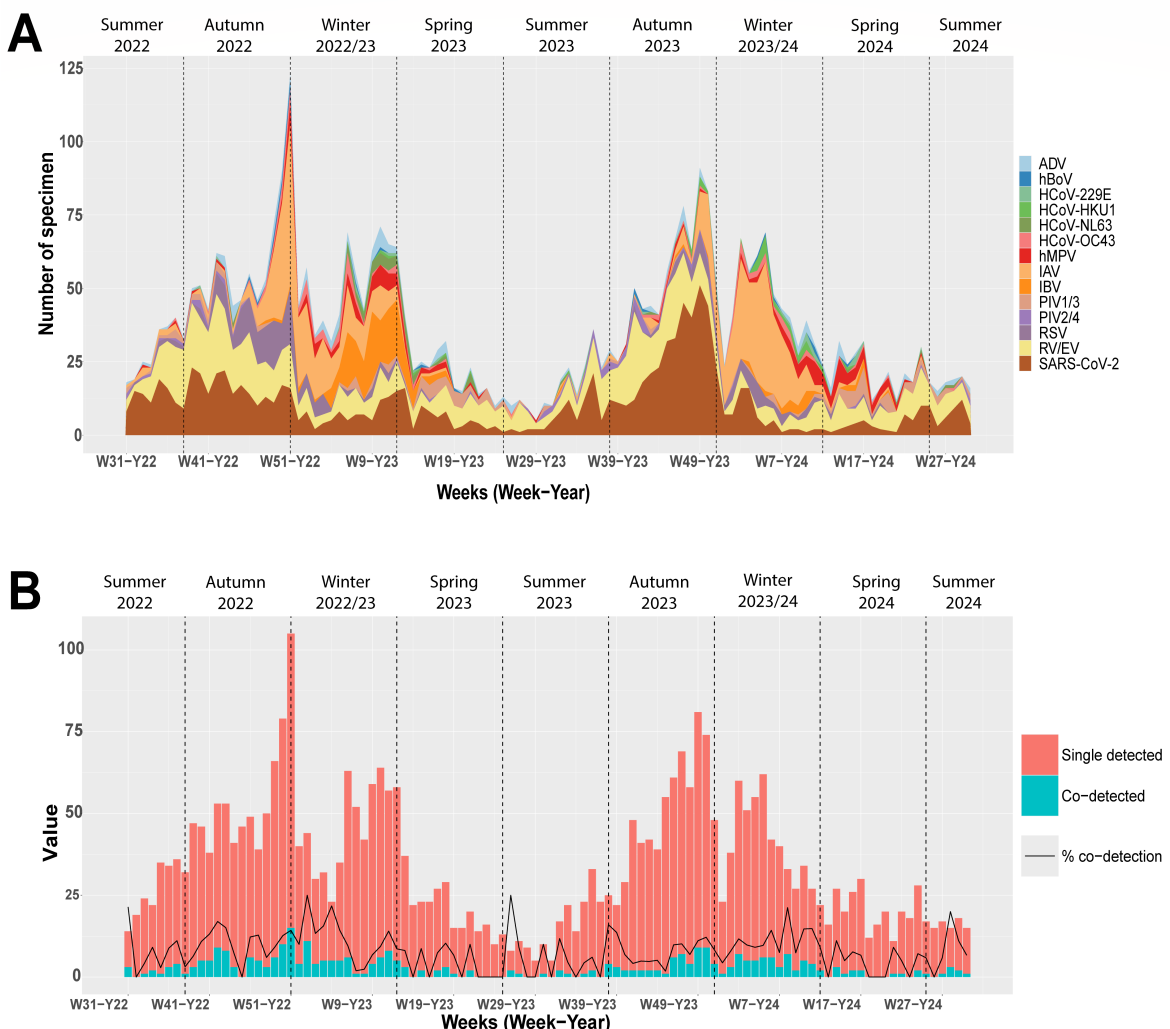
Co-circulation of respiratory viruses during the post-pandemic period. (**A**) Weekly number of clinical specimens, analyzed by the NRCI from week 31 2022 to week 30 2024, positive for each virus or group of viruses. Each colored band represents the number of detections for a specific virus, with the total height corresponding to the sum of all detections per week. The thickness of each band reflects each virus’s relative contribution. (**B**) During the same period, “value” in the Y axis corresponds to the following: weekly number of samples with single (orange bars) and multiple (blue bars) detections and the percentage of samples positive to more than one virus (“co-detection” represented by a solid line). W: week. Y: year. Of note, the data presented in panel **A** were also used in the NRCI annual reports (https://www.hug.ch/laboratoire-virologie/saisons-precedentes). Seasons are indicated on the top of each panel.

This study included data from the analysis of 5,369 nasopharyngeal swab samples originating from 2,466 (46%) male and 2,903 (54%) female cases with age ranging from 0 to 98 years (median 42Y). Among the tested samples, 3,518 (66 %) were positive for at least one respiratory virus (Table 1) with a roughly comparable distribution by age and sex. In both seasons, a similar number of samples were analyzed (*N* = 2,685 and *N* = 2,684 in 2022/2023 and 2023/2024, respectively), but the percentage of samples positive for any pathogen was higher in the first season (68.3%) than in the second (62.7%). Fig. 1A shows that respiratory viruses were detected throughout the 2 years, with higher prevalence in winters, as expected. In this period of the year, the circulation of respiratory viruses was dominated by influenza viruses (except the first peak of the second year, which was dominated by SARS-CoV-2). In line with previous surveillance studies, influenza showed two distinct peaks in both seasons. In both seasons, only influenza A was detected during the first peak. Influenza B was detected during the second peak in both seasons but was dominant only in the second peak of the first season (2022–2023). During both seasons, the maximum number of viruses was detected in December (*N* = 105 in W51/2022 and *N* = 81 in W49/2023) (Fig. 1A and B). RSV, hMPV, and seasonal coronaviruses were mainly detected during the influenza season (week 40 to week 20). Conversely, RV/EV and SARS-CoV-2 were circulating throughout the year and constituted the most prevalent viruses outside of the influenza season (Fig. 1A; Fig. S1A).

**TABLE 1:** Distribution of the study cohort samples

		Number of samples	% of total number of samples
Total number of tested samples		5,369	100
Distribution by sex			
Female		2,903	54.07
Distribution by age			
Children (0–16Y)	Total	828	15.42
	Younger children (0–2Y)	259	4.82
	Older children (>2Y)	569	10.60
Adults (>16Y)	Total	4,541	84.58
	Middle-age adults (<= 65Y)	3,577	66.62
	Older adults (>65)	964	17.95

		Number of samples	% of positive samples
Total number of positive samples		3,518 (66% of total number of samples)	100
Distribution by sex			
Female		1,876	53.33
Distribution by age			
Children (0–16Y)	Total	621	100.00
	Younger children (0–2Y)	215	17.65
	Older children (>2Y)	406	6.11
Adults (>16Y)	Total	2,897	11.54
	Middle-age adults (<= 65Y)	2,246	82.35
	Older adults (>65)	651	63.84

		Number of samples	% of positive samples
Total number of positive samples		3,518 (66 % of total number of samples)	100
Single detection			
One virus		3,204	91
Co-detection			
Two viruses		289	8
Three viruses		23	1
Four viruses		2	<<<1
Total number of co-detections (>1 virus)		314	9

### Co-detection of respiratory viruses

**Fig 2:**
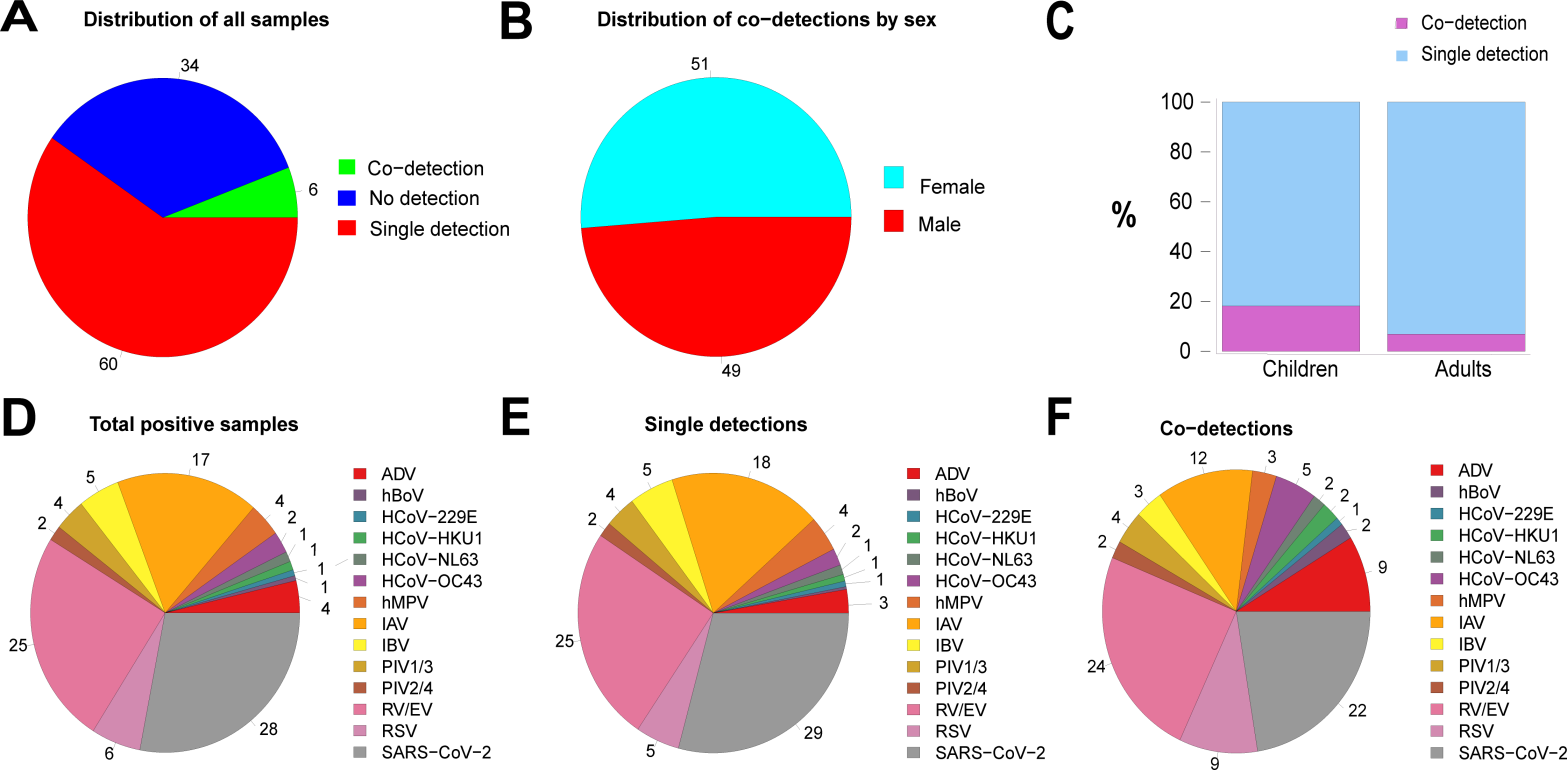
Clinical specimens’ distribution according to detections, gender, age, and virus. (**A**) Proportion of positive (including single and multiple detections) and negative respiratory clinical specimens received and analyzed at the NRCI during the study. (**B**) Prevalence of samples with co-detection in males and females. (**C**) Prevalence (in percentage) of co-detections in children (0–16 years) and adults (>16 years). (**D**) Prevalence of respiratory viruses in total number of positive samples. (**E**) Prevalence of respiratory viruses in samples with single detection. (**F**) Prevalence of respiratory viruses in samples with multiple (co) detections. Details (in numbers) of data displayed in panels **D, E, and F** are provided in Table 2 in columns 2, 3, and 4, respectively.

The study of respiratory virus co-occurrence was based on the prevalence of samples where multiple viruses were detected in the same clinical specimens by (RT-)PCR. Co-detection was found in 9% of the positive samples (Table 1; Fig. 2A) and occurred throughout the year without a clear seasonal pattern. The number of co-detections was proportional (Spearman’s R^2^ = 0.76 and *P* < 0.0001) to the total number of positive samples (Fig. S1B), varying from 0, in several weeks mostly in summer, to a maximum of 25% in W1/Y23 and W26/Y23 during the first season and of 21% during the W8/Y24 of the second season (Fig. 1B). The proportion of co-detection was comparable between males, 49%, and females, 51%, (Fig. 2B). However, co-detections were significantly more frequent in children (18.2%) compared to adults (7%) (OR = 2.98; 95%CI = 2.30–3.85; *P* < 0.0001) (Fig. 2C). Age-related patterns in the sample distribution exhibited relatively little variation for co-detection samples, except in early childhood where their number was increased (Fig. S2).

**TABLE 2:** Total number of viral pathogens identified in single and co-detection and their statistical association with co-detection^*a*^

Virus	Total number of positive samples	Samples with single detections	Samples with co-detections				Fisher's exact test				
			All	Two viruses	Three viruses	Four viruses	Estimated OR	Lower CI	Upper CI	Adjusted *P*-value	Significance
ADV	144	87	57	48	8	1	7.94	5.45	11.50	1.24E-23	****
hBoV	22	10	12	8	2	2	12.67	4.97	33.03	1.39E-07	****
HCoV-229E	27	21	6	5	1	0	2.95	0.97	7.63	0.033,298	*
HCoV-HKU1	39	25	14	11	3	0	5.93	2.82	12.00	5.88E-06	****
HCoV-NL63	46	36	10	8	2	0	2.89	1.27	6.02	0.009,586	**
HCoV-OC43	96	63	33	28	5	0	5.85	3.65	9.23	5.5E-12	****
hMPV	151	132	19	18	1	0	1.50	0.86	2.48	0.117217	ns
IAV	661	589	72	65	7	0	1.43	1.07	1.89	0.015721	*
IBV	193	172	21	17	4	0	1.34	0.80	2.13	0.240711	ns
PIV1/3	144	119	25	21	3	1	2.24	1.37	3.54	0.001536	**
PIV2/4	67	55	12	10	2	0	2.52	1.25	4.74	0.010009	*
RV/EV	958	799	159	145	12	2	3.09	2.42	3.93	1.53E-19	****
RSV	230	168	62	57	5	0	4.44	3.18	6.16	2.93E-16	****
SARS-CoV-2	1071	930	141	125	14	2	2.14	1.68	2.72	1E-09	****

^*a*^ The odds ratios (OR) were calculated using Fisher’s test. CI: 95% confidence interval. For multiple testing, *P*-values were adjusted using the false discovery rate (FDR) method. The statistical significance was assessed by calculating the *P*-values: ≥0.05 (ns),<0.05 (*), <0.01(**), <0.001(***), and <0.0001 (****).

Enteroviruses, RV/EV, (24%) and SARS-CoV-2 (22%) were the most prevalent (Table 2; Fig. 2F) in samples with more than one virus. However, the proportion of viruses detected between single and co-detection varied (Fig. 2D through F). Among the tested respiratory viruses, all could be co-detected with additional respiratory viruses in the same patient sample (Table 2), with most of them being significantly associated with higher prevalence in co-detections (Table 2). This includes some of the most frequently detected viruses, such as RV/EV (OR = 3.09; 95% CI= 2.42–3.93; *P* < 0.0001), RSV (OR = 4.44; 95% CI = 3.18–6.16; *P* < 0.0001), and SARS-CoV-2 (OR = 2.14; 95% CI = 1.68–2.72; *P* < 0.0001) and also ADV (OR = 7.94; 95% CI = 5.45–11.50; *P* < 0.0001) and hBoV (OR = 12.67; 95% CI = 4.97–33.03; *P* < 0.0001), which were detected less often. Influenza viruses showed low or no association with higher prevalence in co-detections (IAV: OR = 1.43; 95% CI = 1.07–1.89; *P* < 0.05. IBV: OR = 1.34; 95% CI = 0.80–2.13; *P* > 0.05).

The majority (91%) of samples with multiple detections were positive for two viruses (dual detections, Table 1). Triple detection was observed in 23 samples, and quadruple detection was seen only in 2 samples during the first season (2022/2023). SARS-CoV-2, RV/EV, hBoV, and ADV were most often detected in samples with three or four viruses simultaneously. More than half of hBoV-positive samples presented co-detections (Table 2).

**Fig 3:**
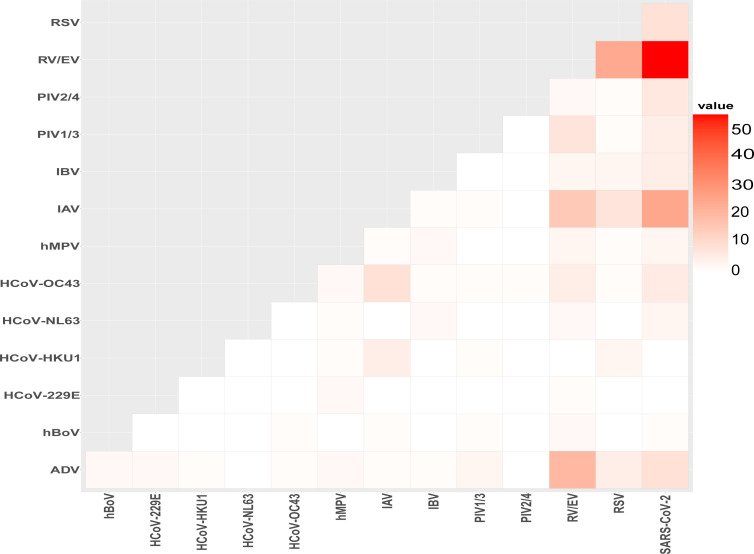
Prevalence of viruses and their associated co-detected viruses. Heatmap presenting the absolute number of samples positive for each pair of viruses in co-detection.

Some combinations of viruses were observed more frequently than others (Fig. 3). For instance, RV/EV is frequently co-detected with ADV, IAV, PIVs, and especially with SARS-CoV-2. The latter is also often found with ADV, RSV, PIVs, hMPV, HCoV-OC43, and HCoV-NL63. Common cold HCoV-OC43 and -HKU1 were also among the most prevalent in co-detections involving IAV.

Altogether, these data show that, although subtle, co-detections happened throughout the year and were more frequent in children. Their occurrence is likely virus-specific, even though they are not necessarily dependent on viral predominance.

### Viral load in single versus co-detection of respiratory viruses

As virus-virus interaction can impact viral replication and shedding, we further investigated the viral load (VL, based on CT values) in samples with a single virus detection versus samples with co-detection of two virus species. Only IAV, IBV, SARS-CoV-2, and RSV as the most prevalent and clinically relevant respiratory viruses were included in this analysis. Despite variability, the overall CT value distribution (including both single and co-detections) was comparable for all these viruses, with medians of 24.2, 22.5, 21.5, and 23.4, respectively (Fig. S3A through D).

**Fig 4:**
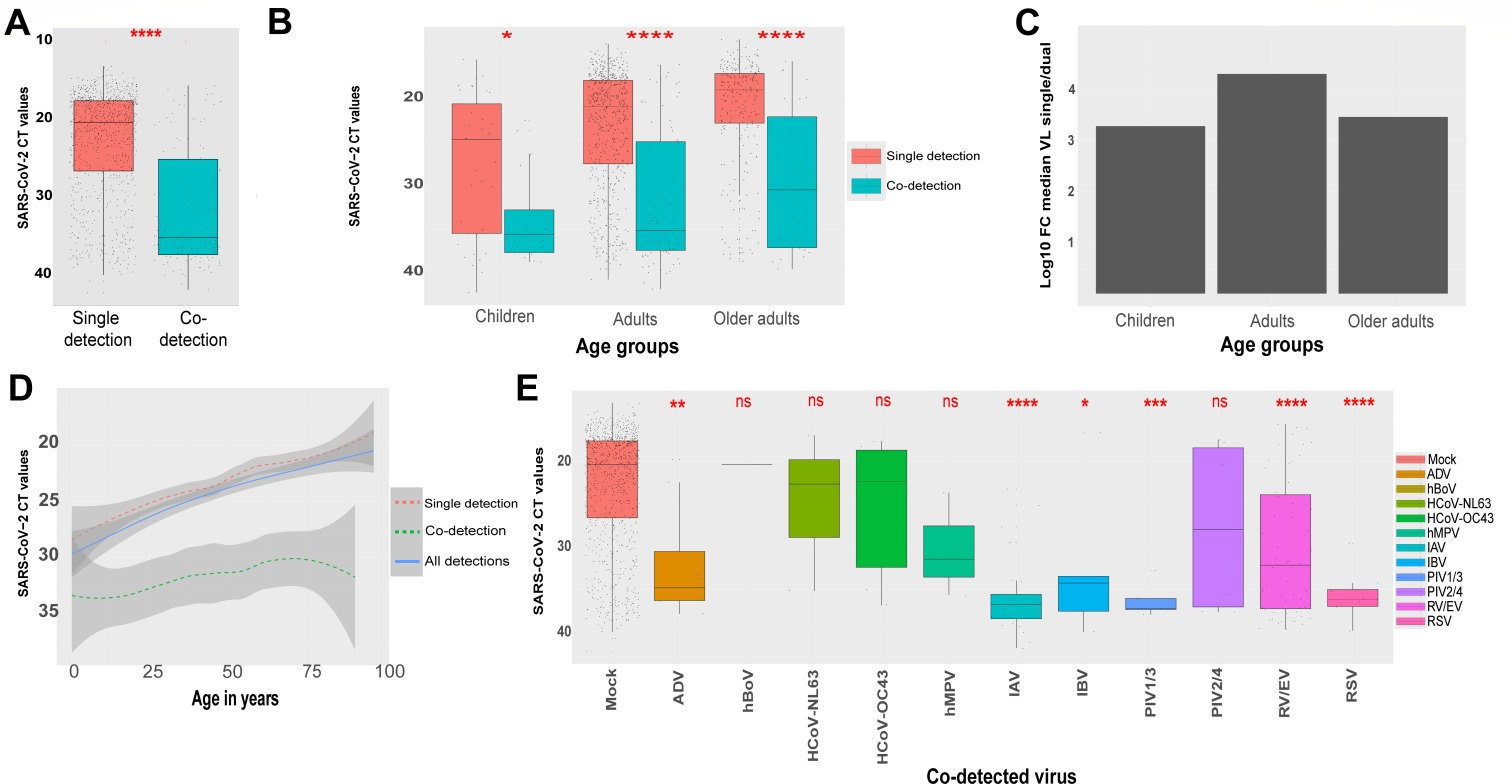
SARS-CoV-2 viral loads in single and dual detections. (**A**) Distribution of SARS-CoV-2 CT values in single versus multiple detections. (**B**) Distribution of SARS-CoV-2 CT values in single versus multiple detections, in each age group, children (0–16 years), adults (16–65 years), and older adults (>65 years). (**C**) Fold decrease of SARS-CoV-2 VL (viral Load) in single relative to co-detections for each age group. This FC is represented in decimal logarithmic and was calculated from the difference of CT medians from single and co-detections. (**D**) Distribution of SARS-CoV-2 CT values according to the patient’s age in total number of positive samples versus samples with single and co-detections. (**E**) Distribution of SARS-CoV-2 CT values in samples with co-detection, depending on the co-detected virus and in comparison to CT values from SARS-CoV-2 single detections (mock). To better reflect the viral load, which is inversely proportional to CTs, all CT axes have been reversed. Statistical comparison of viral loads based on CT values was performed using the Wilcoxon test. Statistical significance was shown by *P*-values: >= 0.05 (ns), <0.05 (*), <0.01(**), <0.001(***), and <0.0001 (****).

When comparing single versus co-detection in samples that tested positive for SARS-CoV-2, median CT values were significantly higher (corresponding to lower VL) in samples presenting co-detections (median CT of 35.3) in comparison to single (median CT of 20.5) detections (Fig. 4A; Fig. S3E). This significant difference was observed in all age groups, including children (younger than 16Y), adults (between 16 and 65Y), and older adults (older than 65Y), being more pronounced in adults (Fig. 4B). Viral load differences (in fold change [FC], calculated based on CT value medians of each age group from co- relative to single detection) were equal to 1.91E03 for children, 2.02E4 for adults, and 2.90E3 for older adults (Fig. 4C). The overall distribution of SARS-CoV-2 CT values showed a very weak but significant correlation (Spearman’s R = −0.259 and *P* < 0.001) between viral loads and age (Fig. 4D, solid line). The same pattern is more observed in single than co-detection (Fig. 4D, dashed lines). Compared to single detections, SARS-CoV-2 viral load was significantly lower in the presence of RV/EV, RSV, influenza viruses, PIV1–3, and adenoviruses (Fig. 4E). Of note, SARS-CoV-2 was barely co-detected with hBoV (*N* = 1), HCoV-NL63 (*N* = 3), and hMPV (*N* = 3).

**Fig 5:**
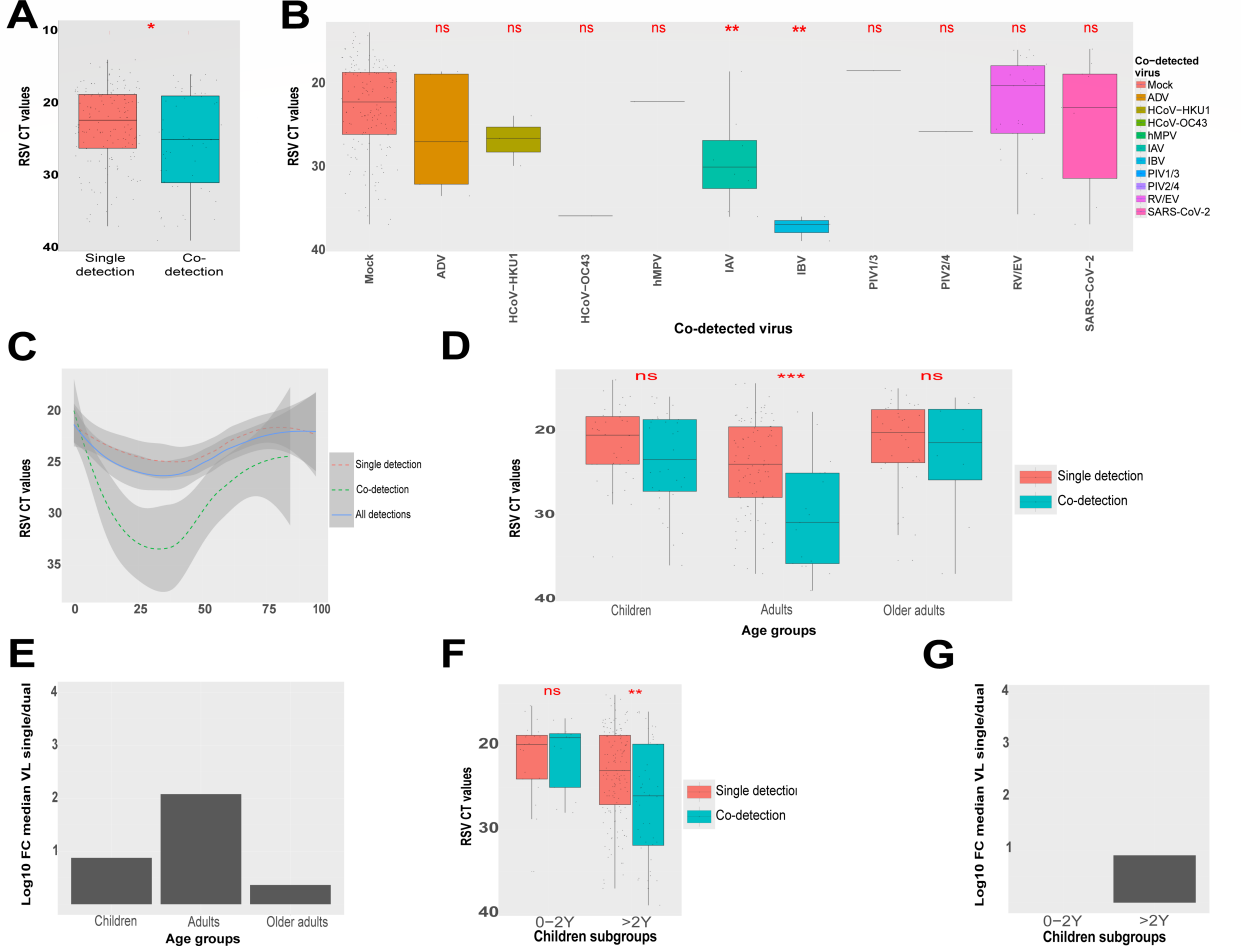
RSV viral loads in single and dual detections. Distribution of RSV CT values in single versus multiple detections. (**A**) Distribution of RSV CT values in single versus multiple detections. (**B**) Distribution of RSV CT values in samples with co-detection, depending on the co-detected virus and in comparison to CT values from RSV single detections (mock). (**C**) Distribution of RSV CT values according to the patient’s age in total number of positive samples versus samples with single and co-detections. (**D**) Distribution of RSV CT values in single versus multiple detections in each age group, including children (0–16 years), adults (16–65 years), and older adults (>65 years). (**E**) Fold decrease of RSV 2 VL (viral load) in single relative to co-detections for each age group. This FC is represented in decimal logarithmic and was calculated from the difference of CT medians from single and co-detections. (**F**) Distribution of RSV CT values in single versus multiple detections in pediatric population that was split into two subgroups of children: younger than 2 years and between 2 and 16 years. (**G**). Fold decrease in RSV viral load in single relative to co-detections for each age group of children, as defined in panel **F**. Fold change is calculated and represented as explained in panel **E**. To better reflect the viral load, which is inversely proportional to CTs, all CT axes have been reversed. Statistical comparison of viral loads based on CT values was performed using the Wilcoxon test. Statistical significance was shown by *P*-values: >= 0.05 (ns), <0.05 (*), <0.01(**), <0.001(***), and <0.0001 (****).

Although to a lower extent, a slight significant decrease in RSV VL was also observed in the context of co-detection (median CT 25) in comparison to single (median CT 22.35) detection (Fig. 5A; Fig. S3F). Even if RSV is mainly detected with RV/EV and SARS-CoV-2, RSV VL decrease was only significant in the co-presence of influenza viruses (Fig. 5B). Seasonal HCoVs (only 3 HCoV-HKU1 and 1 HCoV-OC43), ADV (*N* = 5), PIVs (1 PIV1-3 and 1 PIV2-4), and hMPV (*N* = 1) were absent to barely present in samples with co-detection.

Splitting samples by age groups showed that VL difference was statistically significant only in adults younger than 65 years, as shown in Fig. 5C and D. CT value distribution by age showed higher VL in very young (0–2Y) individuals and older adults (>65Y), compared to older infants and younger adults (Fig. 5C). The most U-shaped age-dependent curve of CTs was observed in the case of samples with co-detection. In line with this observation, RSV VL tends to be lower (119-fold decrease) in the adult group in the presence, compared to absence (Fig. 5D and E), of a second respiratory virus. In children, significant VL decrease in the context of co-detection was only observed in samples collected from patients older than 2 years (8-fold VL decrease in co-detection versus single detection) (Fig. 5F and G).

**Fig 6:**
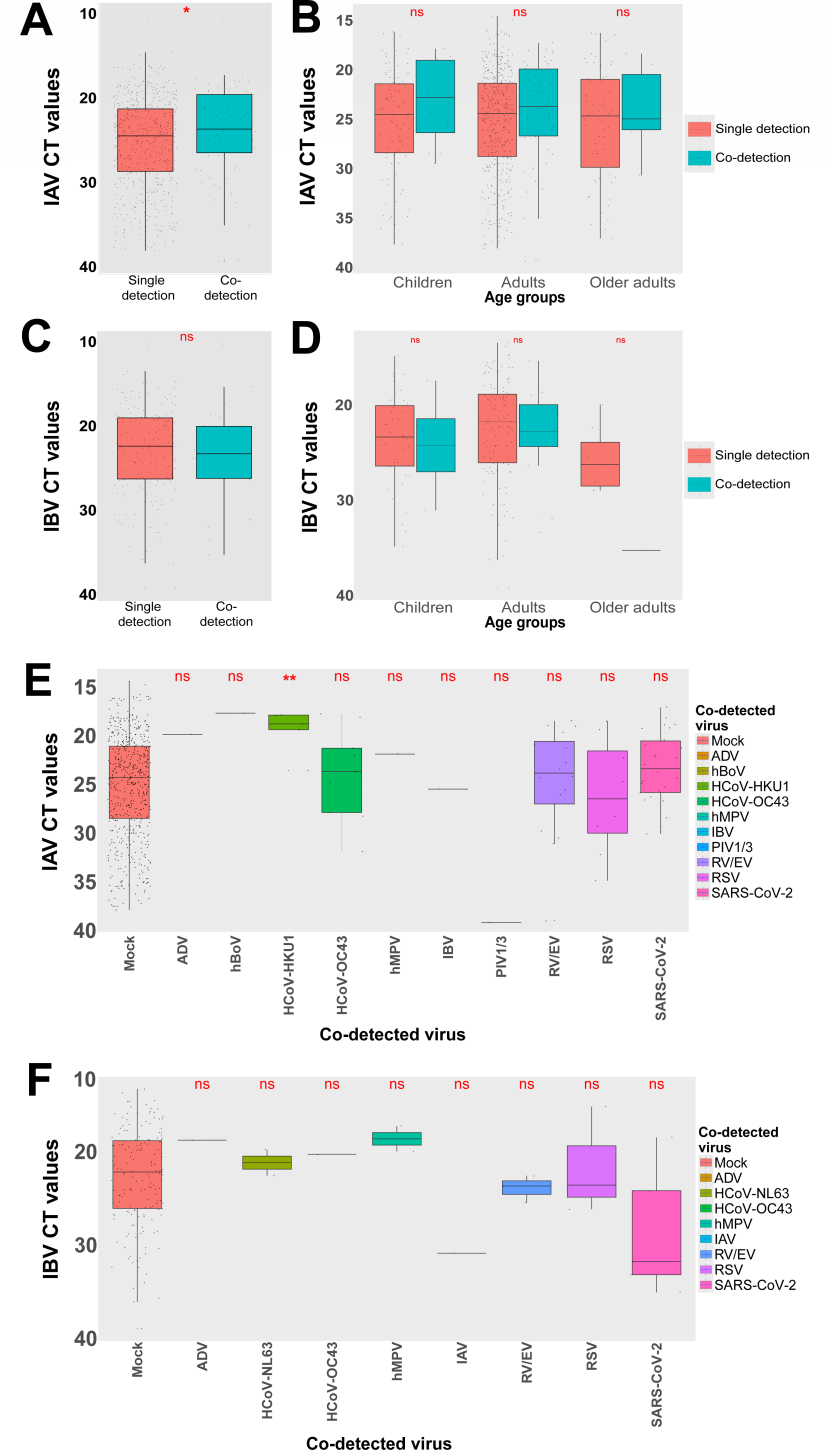
IAV and IBV viral loads in single and dual detections. (**A**) Distribution of IAV CT values in single versus multiple detections. (**B**) Distribution of IAV CT values in single versus multiple detections, in each age group, children (0–16 years), adults (16–65 years), and older adults (>65 years). (**C**) Distribution of IBV CT values in single versus multiple detections. (**D**) Distribution of IBV CT values in single versus multiple detections, in each age group, children (0–16 years), adults (16–65 years), and older adults (>65 years), (**E**) Distribution of IAV CT values in samples with co-detection, depending on the co-detected virus and in comparison to CT values from IAV single detections (mock). (**F**) Distribution of IBV CT values in samples with co-detection, depending on the co-detected virus and in comparison to CT values from IAV single detections (mock). To better reflect the viral load, which is inversely proportional to CTs, all CT axes have been reversed. Statistical comparison of viral loads based on CT values was performed using the Wilcoxon test. Statistical significance is shown by *P*-values: >= 0.05 (ns), <0.05 (*), <0.01(**), <0.001(***), and <0.0001 (****).

Unlike RSV and SARS-CoV-2, no VL decrease was observed in the presence, compared to absence, of a second virus in case of influenza viruses in all age groups (Fig. 6A through D; Fig. S3B). IAV showed even a significant but weak increase (1.74-fold increase) in VL in the context of co-detection (Fig. 6A). This increase was particularly significant and substantial in the co-presence of HCoV-HKU1 (45.25-fold increase) (Fig. 6E). IBV VL was unchanged, regardless of patient ages and the nature of the second virus (Fig. 6C, D and F; Fig. S3H). The prevalence of co-detection of IAV and IBV with other respiratory viruses was zero to very low, except for RV/EV, HCoV-OC43 (only for IAV), RSV, and SARS-CoV-2 (Fig. 3, 6E and F).

In summary, these findings showed that viral loads of SARS-CoV-2 and RSV, but not influenza viruses, were lower in the presence of a second respiratory virus in a virus- and age-dependent fashion. Of note, no season-dependent trend in CT value distributions was observed in samples with either single or multiple detections for SARS-CoV-2, RSV, IAV, or IBV (data not shown).

## DISCUSSION

Viral co-infection *in vitro*, or co-detection in clinical samples *in vivo*, is rarely investigated. From a clinical point of view, the impact of viral co-infections is not well understood, and even less the impact of virus-virus interaction on the epidemiology of respiratory viruses.

Co-detections can occur more often during periods of large epidemics or pandemics and might have an impact on the endemicities of respiratory viruses. Several studies have recently reported co-detections based on data from outpatient settings or hospitalized patients during the first and/or second year of the pandemic (24–28). However, this period corresponds to abnormal and/or diminished circulation of non-SARS-CoV-2 respiratory viruses. Moreover, conclusions from hospitalized patients are biased toward patients with severe clinical presentations and/or underlying conditions. In our work, we decided to study respiratory viral co-detections in the post-pandemic context, where the circulation of viruses returned to pre-pandemic levels. The strength of our study is that data were collected in a systematic way during medical consultations by Sentinella medical doctors across the whole of Switzerland from outpatients defined by ILI/ARI and tested with the same diagnostic assays over the whole study period. Despite the difference in populations and time frames, we found, like in previous studies (25, 28), that the overall proportion of co-detections was low, with some viruses occurring more frequently in combination than others. RV/EV, SARS-CoV-2, and RSV are the most frequent in samples with co-detections, followed by influenza viruses and ADV. The same non-SARS-CoV-2 viruses have been reported to be the most involved in co-detections in a study from patients hospitalized at Geneva University Hospitals in 2011–2012 (9). Unfortunately, testing with the panel of 16 respiratory viruses was only introduced into the Sentinella surveillance in 2020; therefore, no comparisons with the respiratory virus landscape from before the pandemic were possible.

Yet, conducted from an epidemiological point of view, our investigation of co-detections pointed out potential factors likely driving virus-virus associations. It is commonly accepted that airborne viruses, and particularly the enveloped ones, show higher incidence during the winter because of the indoor climate favoring transmission (29). Our data suggested that this enhanced transmission might subsequently favor co-detection occurrence. Despite their low activity, the prolonged shedding of hBoV and ADV (30, 31) also seems to increase their chance to be co-detected with a second, third, or even fourth virus and might explain their significantly enriched proportion in co-, compared to single, infections. Our findings also suggested an age-dependent infection pattern in the context of co-detection. First, co-detections were statistically more common in children compared to adults. A similar observation has been previously reported (28, 32, 33). Second, the studies of viral loads elucidated potential infection impairment that appears dependent not only on the nature of viruses but also on the age of patients. Early and middle-aged adults (16Y–65Y) showed the highest average reduction in viral loads in dual vs single detections. The age-specific differential pattern in co-detections might indicate the involvement of host response in this interference. Older adults, known to have a less efficient immune response and considered at-risk population for VRI (34, 35), showed lower RSV and SARS-CoV-2 inhibition in the context of co-detection. Age-dependent host response in the context of single infections has already been described for respiratory viruses like SARS-CoV-2, influenza viruses, and RSV (36–40) and was related to differential clinical manifestations.

We and other research groups (41–46) had previously placed particular focus on the involvement of host response in viral interference in the context of dual infections. These studies demonstrated, experimentally, that viral interference in the context of multiple infections depends on the capacity of the first virus to trigger an interferon (IFN) response and hence to block a potential superinfection by another virus, provided that the latter is sensitive to innate immune induction. Supporting these translational research findings, we here underlined the possible interference exerted on SARS-CoV-2 and RSV, which are susceptible to IFN, but not influenza viruses, which are high IFN inducers but less susceptible to IFN, in the context of co-infections. From a biological perspective, investigating the mechanisms of virus-virus interference provides crucial insights into viral pathogenesis and immune response modulation. Questions remain regarding the molecular pathways implicated in the age-related disparate virus-virus interaction and might help unveil therapeutical targets against respiratory viruses. Other mechanisms might also be involved in the protection against superinfection like cross-immunity between genetically related viruses like hMPV, RSV, and PIV (47) and competition in using host resources for viral replication (48, 49).

Clinically, interference between respiratory viruses could influence disease severity and patient outcomes. Whether coinfection leads to mitigated or more severe illness is still debated, as reviewed in reference 50. Lansbury et al. (24) reported in a systematic review and meta-analysis about respiratory co-infection involving SARS-CoV-2 that bacterial, compared to viral, co-detections were associated with an increased proportion of hospitalization and admission to the intensive care unit. Of note, in our study, we did not assess bacterial pathogens and had no access to the day(s) of symptom onset nor the disease severity related to the patients, and no asymptomatic individuals were included. Only sex and age anonymized data could be analyzed; we could not investigate more aspects, such as the sequence of infections, the number of episodes per patient, the incubation time between the two infections, and the time since symptom onset, that might be related to co-detections and/or have an impact on viral loads. Although no housekeeping host gene was included for sample-quality normalization (51), extraction efficiency was monitored using the spiked internal control, CDV. Co-detection was assessed by testing the presence of viral genomes in clinical samples, which cannot distinguish between co-infections and single active infections along with remnants from previous infections, a common problem when assessing clinical specimens.

The examination of viral loads was based on CT values obtained by semi-quantitative viral genome amplification, the gold standard method for viral detection in diagnostic purposes. Although not reflecting the viral infectiousness (which is an important factor for transmissibility) and might be affected by factors such as sample quality and extraction efficiency, CTs provide a good approximation of the virus yield present in the sample. In line with our findings, an Ecuadorian study recently reported less SARS-CoV-2 viral copy number in co-, compared to single, infections by SARS-CoV-2 (27). In addition to the reduced viral load, a negative association between viruses could also be statistically assessed by the absence or low frequency of co-detection events, as it has been reported for the pandemic influenza virus in 2009 and rhinoviruses (52, 53). In our study, the higher IAV VL in the presence of HCoV-HKU1 might suggest a positive association between the two viruses, which deserves to be confirmed by more clinical and *in vitro* data.

Altogether, our study constitutes a large description of the new post-COVID-19 pandemic VRI picture in Switzerland. It also emphasizes the phenomenon of viral respiratory co-occurrence and sheds light on the importance of such community-based surveillance, beyond the monitoring of respiratory disease burden, to generate hypotheses based on real-world evidence, for a better understanding of the viral biology and the virus-host interplay upon infection.

## Data Availability

In accordance with the strict conditions outlined in the data transfer and use agreement with the Federal Office of Public Health, only aggregated data from this study may be presented. No additional data will be made available in any repository.

## SUPPLEMENTAL MATERIAL

Supplemental material spectrum.03088-25-s0001.docx Table S1; Fig. S1 to S3.

ASM does not own the copyrights to Supplemental Material that may be linked to, or accessed through, an article. The authors have granted ASM a non-exclusive, world-wide license to publish the Supplemental Material files. Please contact the corresponding author directly for reuse.
