# Supplementary material for: Swiss community-based surveillance of respiratory virus co-occurrence in 2022–2024
Source: Microbiol Spectr. 2026 Mar 30;14(5):e03088-25. doi: 10.1128/spectrum.03088-25 (PMC13141836; doi:10.1128/spectrum.03088-25)
Supplement: Supplemental material — Table S1; Fig. S1 to S3. [file spectrum.03088-25-s0001.docx]

## Supplementary tables

**Supplementary table S1.** List of custom primers and probes used for the detection of respiratory viruses in this study. * RV RT-PCR assay cross detects with lower sensitivity the non-rhinovirus EVs, from the same family of picornaviruses. **Two RT-PCR assays are used to detect, in pairs, the 4 PIVs. These assays can distinguish PIV-1 and -3 from PIV-2 and -4 but cannot specifically differentiate PIV-1 from -3 and -2 from -4. ^a^: HUG, Geneva University Hospitals. ^b^: lna, locked nucleic acid

| **Pathogen** | **Primer/Probe name** | **Primer/Probe sequence** | **Final concentration (nM)** | **Reference** |
| --- | --- | --- | --- | --- |
| hBoV | bocafor | CACTGGCAGACAACTCATCACA | 600 | Modified from (1, 2) |
|  | bocarev | GATATGAGCCCGAGCCTCTCT | 600 |  |
|  | bocaprob | [FAM]AGCAGGAGCCGCAGCCCGA[TAMRA] | 100 |  |
| ADV | ADVfor(AQ2) | GCCCCAGTGGTCTTACATGCACATC | 600 | Modified from (3) |
|  | ADVrevnew60 | GVGCCACGGTGGGGTTTCTAAACTT | 600 |  |
|  | ADVpro | [FAM]TGCACCAGACCCGGGCTCAGGTACTCCGA[BHQ1] | 100 |  |
| IAV | InfA-CDC For | GACCRATCCTGTCACCTCTGAC | 900 | From WHO(4) |
|  | InfA-CDC Rev | AGGGCATTYTGGACAAAKCGTCTA | 900 |  |
|  | InfA duplex Pro CDC | [FAM]TGCAGTCCTCGCTCACTGGGCACG[BHQ1] | 200 |  |
| IBV | BcdcFor | TCCTCAAYTCACTCTTCGAGCG | 900 | Modified from CDC(5, 6) |
|  | BcdcRev | CGGTGCTCTTGACCAAATTGG | 900 |  |
|  | Bcdc duplex Pro | [ATTO 647N]CCAATTCGAGCAGCTGAAACTGCGGTG[BHQ2] | 200 |  |
| RSV | RSVAB For | CATGGARAAGTTTGCWCCTGAAT | 300 | In-house HUG^a^ |
|  | RSVAB Rev | GCCYTTTATTGATTCTAGGAAT | 300 |  |
|  | RSVAB duplex Pro | [FAM]CATGGAGAAGAYGCA[MGB] | 200 |  |
| hMPV | hMPV_N_GeF | CATAYAARCATGCTATATTAAAAGAGTCTCA | 300 | Modified from (7, 8) |
|  | hMPV_N-Ge R2 | CCTATYTCWGCAGCATATTTGTARTCAG | 300 |  |
|  | hMPV_N-Ge duplex P2 | [ATTO 647N]CAACHGCAGTRACACCYTCATCATTRCA[BHQ2] | 100 |  |
| RV* | PicoFor | AGCCTGCGTGGCKGCC | 900 | Modified from (9) |
|  | fwd_PanpicoLee | CYlnaAGCClnaTGCGTGG ^b^ | 900 |  |
|  | PicoRev | GAAACACGGACACCCAAAGTAGT | 900 |  |
|  | Pico duplex Pro | [ATTO 647N]CTCCGGCCCCTGAATGYGGCTAA[BHQ2] | 100 |  |
| PIV-1 / -3** | P1-3a for | ATCCAAGAGGRGGAATAGA | 600 | Modified from (10) |
|  | P1-3b for | ACCCAAGAGGGGGTATAGA | 600 |  |
|  | P1-3 rev | GTCTCCTTGRACCATTGC | 900 |  |
|  | HPIV1-3pro 1 duplex | [FAM]TCTATTAGTGCAATCCATCTTGCRGCTGTT[BHQ1] | 100 |  |
|  | HPIV1-3pro 3 duplex | [FAM]TCCATAAGTGCAATMCATCTAGCAGCTGTT[BHQ1] | 100 |  |
| PIV-2 / -4** | P2 for duplex | TTATGAARGACAAGGCAATAAG | 750 | In-house HUG^a^ |
|  | P2 rev duplex | TAAATGGATTGGGCATAGG | 750 |  |
|  | P4 for duplex | ATTCATGAARGATAAAGCAATAAG | 750 |  |
|  | P4 rev duplex | CTATTYACATTYGATGGACATTC | 750 |  |
|  | P24 pro duplex | [FAM]AATACACTCATCCAAT[MGB] | 200 |  |
| HCoV-HKU1 | HKU1 for | AATACGGTCTCGGCTTCAAGTG | 600 | In-house HUG^a^ |
|  | HKU1 rev | CCAACTCCTCCGCTGCAT | 600 |  |
|  | HKU1 pro | [FAM]CGCCAGAATTTCGTTGGCTGCTTC[BHQ1] | 200 |  |
| HCoV-229E | 229E for | CAGTCAAATGGGCTGATGCA | 400 | From (11) |
|  | 229E rev | AAAGGGCTATAAAGAGAATAAGGTATTCT | 400 |  |
|  | 229E pro | [FAM]CCCTGACGACCACGTTGTGGTTCA[BHQ1] | 200 |  |
| HCoV-OC43 | OC43 for | CGATGAGGCTATTCCGACTAGGT | 400 | Modified from (11) |
|  | OC43 rev | CCTTCCTGAGCCTTCAATATAGTAACC | 400 |  |
|  | OC43 pro | [ATTO647N]TCCGCCTGGCACGGTACTCCCT[BHQ2] | 200 |  |
| HCoV-NL63 | NL63 for | AAACCTCGTTGGAAGCGTGT | 600 | Modified from (12) |
|  | NL63 rev | CTGTGGAAAACCTTTGGCATC | 600 |  |
|  | NL63 pro | [ATTO647N]ATGTTATTCAGTGCTTTGGTCCTCGTGAT[BHQ2] | 200 |  |
| CDV (internal conntrol) | CDV 1 (for) | GCTACCCAAGAAACCGTCATTG | 900 | In-house HUG^a^ |
|  | CDV 1 (rev) | GCATGGCAGGGACGAGTT | 900 | In-house HUG^a^ |
|  | CDV (probe) | VIC-CGTTCAGGGAGTCCAGGACTACGTCAAC-TAMRA | 200 | In-house HUG^a^ |

## Supplementary figures

**
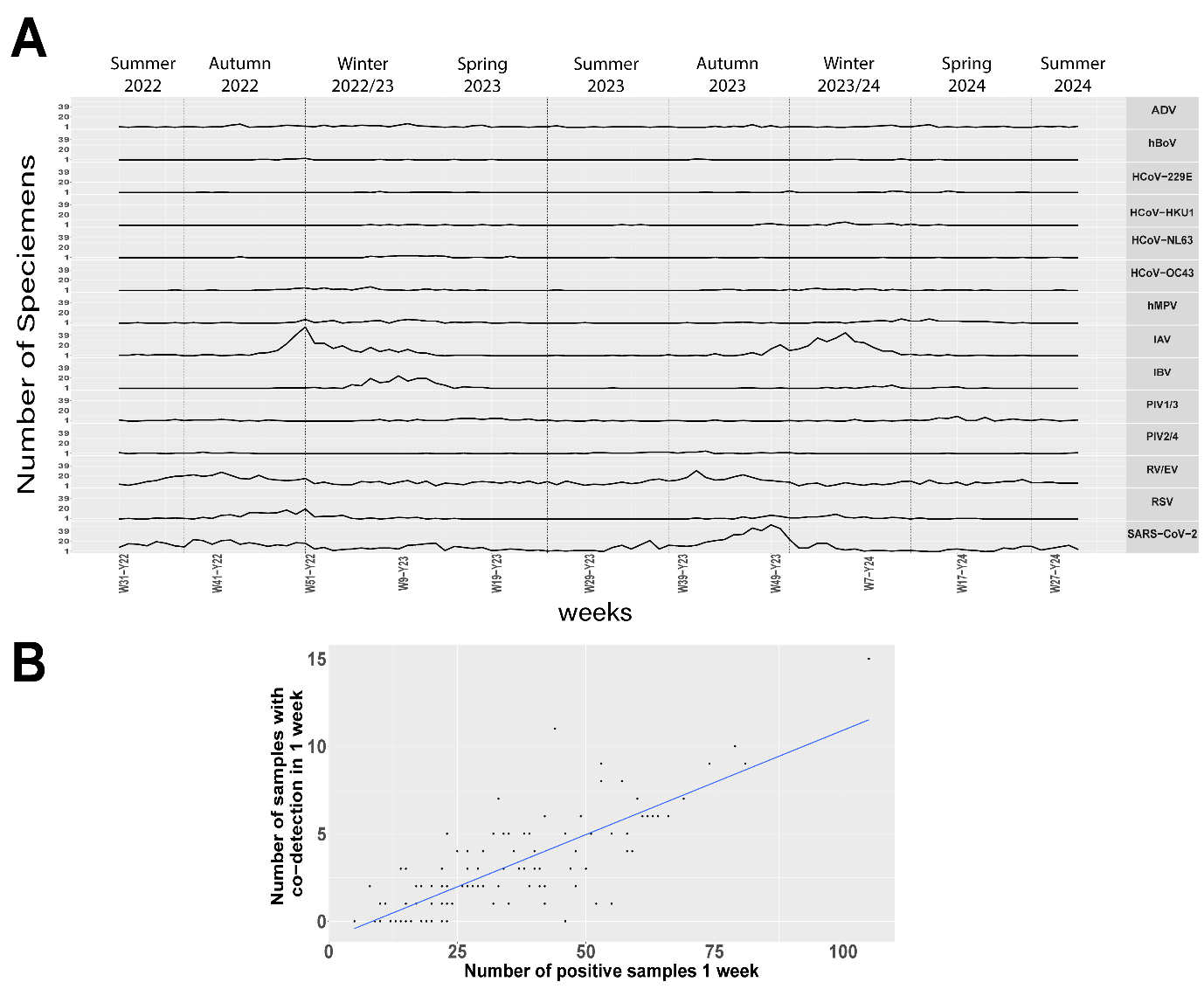
FigS1: Weekly distribution of clinical specimens.** A. weekly number of clinical specimens, analysed by NCRI from week 31 2022 to week 30 2024, positive for each virus or group of viruses. Seasons are indicated on the top of the graph. B. Correlation between the weekly number of positive samples and the weekly number of samples with co-detection.

**
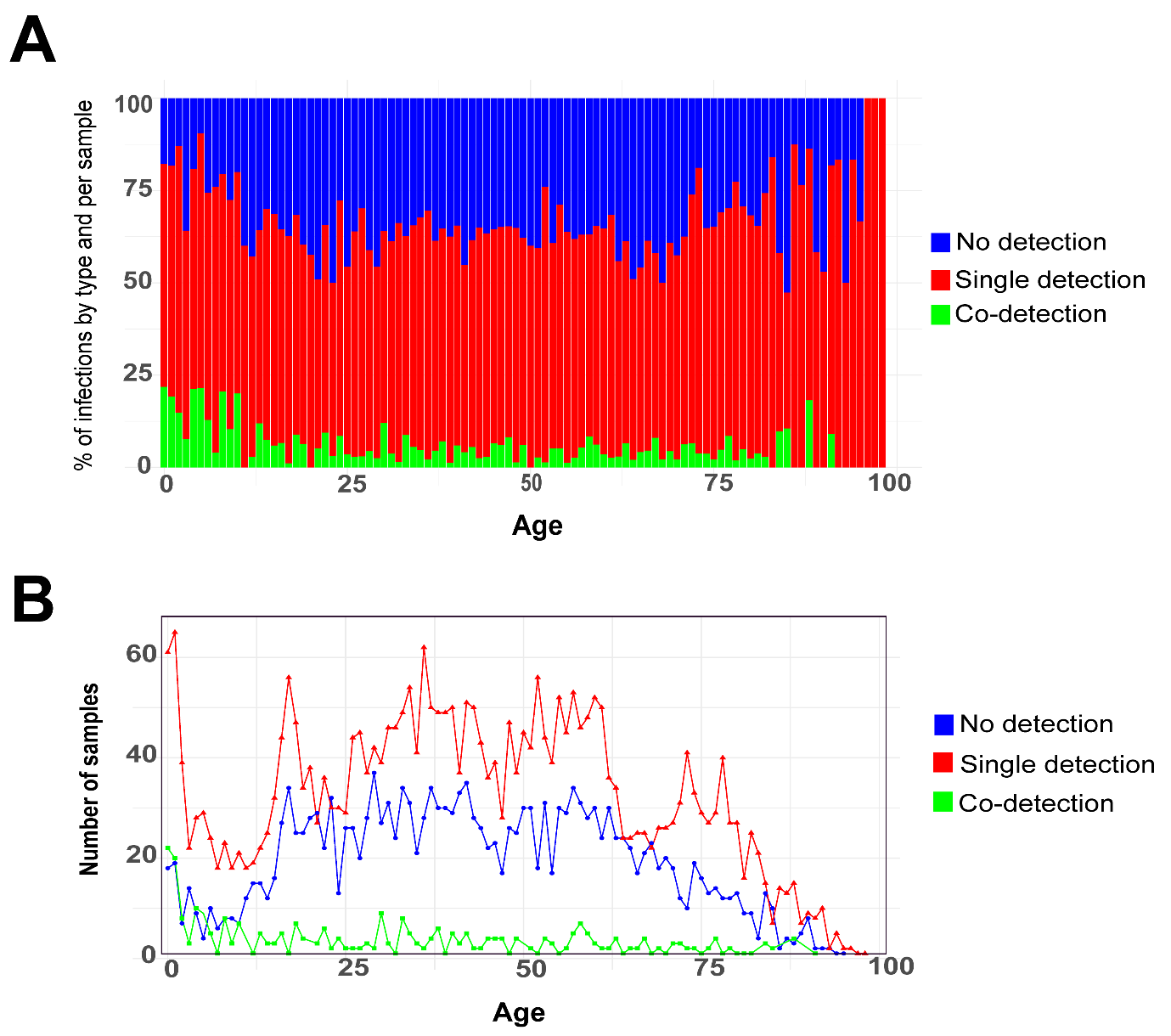
**

**FigS2: Distribution of tested samples by age.** A. Distribution of sample proportion (with no detection, single or co-detection) by age. B. Distribution of the number samples with no, single or multiple detection by age.

**
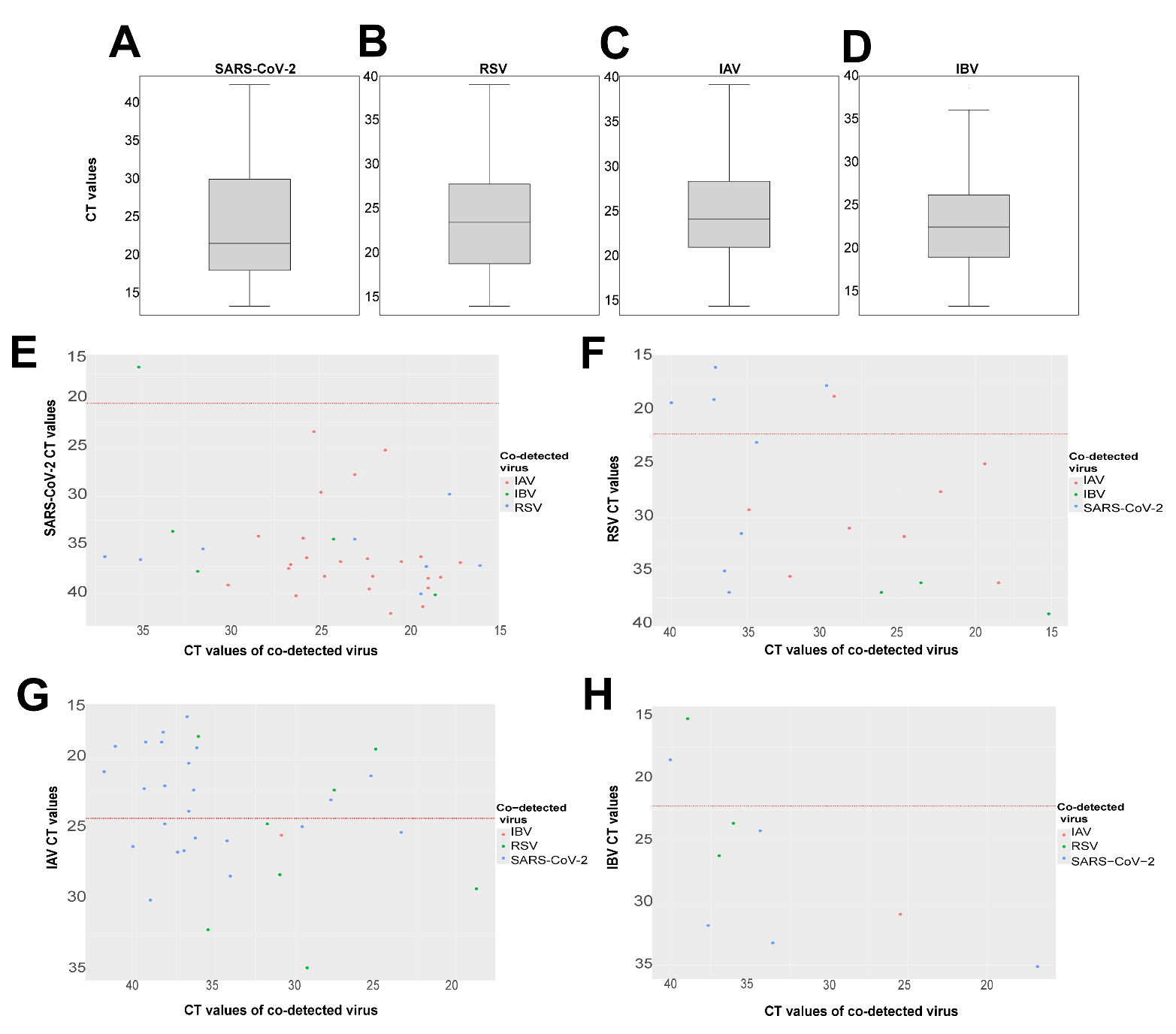
FigS3. Examination of SARS-CoV-2, RSV, IAV and IBV CT values**. A. Distribution of SARS-CoV-2 CT values. B. Distribution of RSV CT values. C. Distribution of IAV CT values. D. Distribution of IBV CT values. E. SARS-CoV-2 CT values, depending on CT values of the co-detected RSV, IAV and IBV. F. RSV CT values, depending on CT values of the co-detected SARS-CoV-2, IAV and IBV. G. IAV CT values, depending on CT values of the co-detected SARS-CoV-2, RSV and IBV. H. IBV CT values, depending on CT values of the co-detected SARS-CoV-2, RSV and IBV. In panels E to H, the CT value medians of single detection by SARS-CoV-2 (E), RSV (F), IAV (G) and IBV (H) are represented by the red dashed line.

**Bibliography**

1. Ligozzi M, Diani E, Lissandrini F, Mainardi R, Gibellini D. 2017. Assessment of NS1 gene-specific real time quantitative TaqMan PCR for the detection of Human Bocavirus in respiratory samples. Mol Cell Probes 34:53–55.

2. Kantola K, Sadeghi M, Antikainen J, Kirveskari J, Delwart E, Hedman K, Söderlund-Venermo M. 2010. Real-Time Quantitative PCR Detection of Four Human Bocaviruses. J Clin Microbiol 48:4044–4050.

3. Heim A, Ebnet C, Harste G, Pring-Akerblom P. 2003. Rapid and quantitative detection of human adenovirus DNA by real-time PCR. J Med Virol 70:228–239.

4. Information for molecular diagnosis of influenza virus. https://www.who.int/teams/global-influenza-programme/laboratory-network/quality-assurance/eqa-project/information-for-molecular-diagnosis-of-influenza-virus. Retrieved 10 January 2025.

5. Shu B, Kirby MK, Davis WG, Warnes C, Liddell J, Liu J, Wu K-H, Hassell N, Benitez AJ, Wilson MM, Keller MW, Rambo-Martin BL, Camara Y, Winter J, Kondor RJ, Zhou B, Spies S, Rose LE, Winchell JM, Limbago BM, Wentworth DE, Barnes JR. 2021. Multiplex Real-Time Reverse Transcription PCR for Influenza A Virus, Influenza B Virus, and Severe Acute Respiratory Syndrome Coronavirus 2. Emerg Infect Dis 27:1821–1830.

6. CDC. 2024. CDC’s Influenza SARS-CoV-2 Multiplex Assay. Influenza (Flu). https://www.cdc.gov/flu/php/laboratories/influenza-sars-cov-2-multiplex-assay.html. Retrieved 10 January 2025.

7. Maertzdorf J, Wang CK, Brown JB, Quinto JD, Chu M, de Graaf M, van den Hoogen BG, Spaete R, Osterhaus ADME, Fouchier RAM. 2004. Real-time reverse transcriptase PCR assay for detection of human metapneumoviruses from all known genetic lineages. J Clin Microbiol 42:981–986.

8. Carr MJ, Waters A, Fenwick F, Toms GL, Hall WW, O’Kelly E. 2008. Molecular epidemiology of human metapneumovirus in Ireland. J Med Virol 80:510–516.

9. Tapparel C, Cordey S, Van Belle S, Turin L, Lee W-M, Regamey N, Meylan P, Mühlemann K, Gobbini F, Kaiser L. 2009. New molecular detection tools adapted to emerging rhinoviruses and enteroviruses. J Clin Microbiol 47:1742–1749.

10. Cordey S, Thomas Y, Cherpillod P, van Belle S, Tapparel C, Kaiser L. 2009. Simultaneous detection of parainfluenza viruses 1 and 3 by real-time reverse transcription-polymerase chain reaction. J Virol Methods 156:166–168.

11. Hammitt LL, Kazungu S, Welch S, Bett A, Onyango CO, Gunson RN, Scott JAG, Nokes DJ. 2011. Added value of an oropharyngeal swab in detection of viruses in children hospitalized with lower respiratory tract infection. J Clin Microbiol 49:2318–2320.

12. Luna LK de S, Panning M, Grywna K, Pfefferle S, Drosten C. 2007. Spectrum of viruses and atypical bacteria in intercontinental air travelers with symptoms of acute respiratory infection. J Infect Dis 195:675–679.
